# Delphi prioritization and development of global surgery guidelines for the prevention of surgical‐site infection

**DOI:** 10.1002/bjs.11530

**Published:** 2020-03-24

**Authors:** National Institute for Health Research Global Research Health Unit on Global Surgery, Dmitri Nepogodiev, Adewale Adisa, Francis Atindaana Abantanga, Adesoji Ademuyiwa, Sohini Chakrabortee, Dhruva Ghosh, James Glasbey, Marie Carmela Lapitan, Ismail Lawani, Mayaba Maimbo, Rachel Moore, Dion Morton, Faustin Ntirenganya, Ahmad Uzair Qureshi, Antonio Ramos‐De la Medina, Stephen Tabiri, Thomas Pinkney, Aneel Bhangu, Adesoji Ademuyiwa, Anthony Adenekan, Abdus‐sami Adewunmi, Adewale Adisa, Maria Lorena Aguilera, Aneel Bhangu, Bruce Biccard, Peter Brocklehurst, Sohini Chakrabortee, Ainhoa Costa, Philip Cotton, Justine Davies, Thomas M. Drake, O. James Garden, Dhruv Ghosh, James Glasbey, Parvez David Haque, Ewen M. Harrison, Jean De La Croix Allen Ingabire, Stephen R. Knight, Marie Carmela Lapitan, Ismail Lawani, Richard Lilford, Mayaba Maimbo, Janet Martin, Luis Hernandez Miguelena, Rohin Mittal, Rachel Moore, Dion Morton, Vanessa Msosa, Syed Asghar Naqi, Dmitri Nepogodiev, Faustin Ntirenganya, Jean Leon Olory‐Togbe, Omar Mohamed Omar, Thomas D. Pinkney, Ahmad Uzair Qureshi, Antonio Ramos‐De la Medina, Hosni Khairy Salem, Martin Smith, Sudha Sundar, Stephen Tabiri, Edwin Yenli, Raul Yepez, Eugene Zoumenou, Francis Atindaana Abantanga, Adesoji Ademuyiwa, Abdus‐sami Adewunmi, Adewale Adisa, Maria Lorena Aguilera, Aneel Bhangu, Bruce Biccard, Peter Brocklehurst, Sohini Chakrabortee, Ainhoa Costa, Thomas M. Drake, Dhruva Ghosh, James Glasbey, Parvez David Haque, Ewen M. Harrison, Jean De La Croix Allen Ingabire, Conor S Jones, Chifundo Kajombo, Stephen R. Knight, Marie Carmela Lapitan, Ismail Lawani, Samuel Lawday, Mayaba Maimbo, Janet Martin, Luis Hernandez Miguelena, Rohin Mittal, Rachel Moore, Dion Morton, Vanessa Msosa, Syed Asghar Naqi, Dmitri Nepogodiev, Faustin Ntirenganya, Martin Nyundo, Jean Leon Olory‐Togbe, Thomas D. Pinkney, Ahmad Uzair Qureshi, Antonio Ramos‐De la Medina, Dione Parreno‐Sacdalan, Hosni Khairy Salem, Martin Smith, Stephen Tabiri, Edwin Yenli, Eugene Zoumenou

## Abstract

**Background:**

Most clinical guidelines are developed by high‐income country institutions with little consideration given to either the evidence base for interventions in low‐ and middle‐income countries (LMICs), or the specific challenges LMIC health systems may face in implementing recommendations. The aim of this study was to prioritize topics for future global surgery guidelines and then to develop a guideline for the top ranked topic.

**Methods:**

A Delphi exercise identified and prioritized topics for guideline development. Once the top priority topic had been identified, relevant existing guidelines were identified and their recommendations were extracted. Recommendations were shortlisted if they were supported by at least two separate guidelines. Following two voting rounds, the final recommendations were agreed by an international guideline panel. The final recommendations were stratified by the guideline panel as essential (baseline measures that should be implemented as a priority) or desirable (some hospitals may lack these resources at present, in which case they should plan for future implementation).

**Results:**

Prevention of surgical‐site infection (SSI) after abdominal surgery was identified as the highest priority topic for guideline development. The international guideline panel reached consensus on nine essential clinical recommendations for prevention of SSI. These included recommendations concerning preoperative body wash, use of prophylactic antibiotics, decontamination of scrub teams' hands, use of antiseptic solutions for surgical site preparation and perioperative supplemental oxygenation. In addition, three desirable clinical recommendations and four recommendations for future research were agreed.

**Conclusion:**

This process led to the development of a global surgery guideline for the prevention of SSI that is both clinically relevant and implementable in LMICs.

## Graphical abstract

A global surgery guideline for the prevention of surgical‐site infection has been developed that is both clinically relevant and implementable in low‐ and middle‐income countries (LMICs). This guideline includes nine essential and three desirable clinical recommendations.

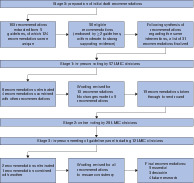

Usable worldwide

## Abstract (es)

**Antecedentes:**

La mayoría de las guías clínicas están desarrolladas por instituciones de países con altos ingresos, con poca consideración respecto a las bases de la evidencia para intervenciones en países con ingresos bajos y medios (*low‐ and middle‐income countries*, LMIC) o a los desafíos específicos que los sistemas de salud de LMIC pueden enfrentarse al implementar las recomendaciones. El objetivo de este estudio fue priorizar los temas para futuras guías quirúrgicas globales y seguidamente desarrollar una guía para el tema categorizado en primer lugar.

**Métodos:**

Un proceso tipo Delphi identificó y priorizó los temas para el desarrollo de las guías. Una vez identificado el tema de máxima prioridad, se seleccionaron las guías existentes más relevantes y se extrajeron sus recomendaciones. Las recomendaciones fueron preseleccionadas si estaban respaldadas por al menos dos guías distintas. Después de dos rondas de votación, las recomendaciones finales fueron acordadas por un panel internacional de expertos en guías. Las recomendaciones finales fueron estratificadas por el panel como esenciales (medidas básicas que deberían implementarse como una prioridad) o deseables (algunos hospitales pueden carecer de estos recursos en este momento, en cuyo caso deberían plantear una implementación futura).

**Resultados:**

El método Delphi identificó la prevención de la infección del sitio quirúrgico (*surgical‐site infection*, SSI) después de la cirugía abdominal como el tema de máxima prioridad para el desarrollo de guías. El borrador inicial de las recomendaciones se desarrolló en base a cinco guías existentes que abordaban la prevención de SSI. Después de dos rondas de votación, el panel internacional acordó una lista de nueve recomendaciones clínicas esenciales y tres deseables. Además, se hicieron cuatro recomendaciones para futuras investigaciones.

**Conclusión:**

Este proceso desarrolló una guía quirúrgica global para la prevención de SSI que es clínicamente relevante y además se puede implementar en los LMICs.

## Introduction

Clinical guidelines produce evidence‐based recommendations that aim to standardize patient care by identifying best practice. Implementation of clinical guidelines into routine practice has the potential to reduce complications, mortality and costs^1^, ^2^, ^3^, ^4^. Despite an increasing trend in clinical guideline production by a variety of bodies^4^, there remains a lack of national or international clinical guidelines designed to meet the needs of patients and health services in low‐ and middle‐income countries (LMICs)^5^. Frequently LMIC clinicians have no alternative to using clinical guidelines published in high‐income countries (HICs) by authors with limited experience of the pathologies and challenges faced in lower‐resource settings. Consequently, guideline implementation in LMICs is inconsistent and the potential benefits are not fully realized.

To increase the likelihood of guidelines being adopted internationally, particular consideration needs to be given to the specific resource and logistical challenges LMIC health systems may face in implementing recommendations^5^. Additionally, given that many existing clinical guidelines include large numbers of recommendations, there is a need for streamlined guidelines which can inform prioritization of implementation of key practical measures.

The aim of this study was to prioritize topics for future global surgery guidelines and then to develop a guideline for the top ranked topic. Development of new clinical guidelines is expensive and time‐consuming. Rather than commissioning entirely new guidelines, the National Institute for Health Research (NIHR) Global Health Research Unit on Global Surgery (GSU) network aimed to build on existing HIC guidelines by adapting these to LMIC settings.

## Methods

### Prioritization of global surgery guideline topics

A three‐round Delphi exercise was conducted among the international NIHR‐GSU network to prioritize topics for global surgery guidelines. In the first round, collaborators proposed guideline topics. In the second round, an international panel shortlisted the top 12 proposed guideline topics. An anonymous online vote by LMIC stakeholders then prioritized the shortlisted topics in the third round. The full methodology for the prioritization exercise is reported in *Appendix* *S2* (supporting information).

### Stage 0: preparation of initial draft recommendations

This highest‐priority guideline was taken forward into development of a full guideline, in keeping with the International Standards for Clinical Practice Guidelines^6^. Adherence to these standards is reported in *Table* *S1* (supporting information). A comprehensive search of PubMed and Google Scholar was completed to identify existing national and international guidelines relevant to the prioritized guideline. In addition, members of the international NIHR‐GSU network were asked to identify any relevant national guidelines. To ensure that contemporaneous evidence informed the global guideline, only guidelines published in the previous 5 years (2013–2018) were eligible. Only English‐language guidelines were considered.

Each recommendation made by the eligible guidelines was extracted into a spreadsheet and coded by intervention to facilitate further analysis. Recommendations were longlisted if they were endorsed by at least two separate guidelines, with at least one of these having reported moderate or strong evidence to support the recommendation (see example in *Table* *S2*, supporting information). Because multiple guidelines frequently made slightly different recommendations regarding the same interventions, similar recommendations were combined to produce a streamlined initial list of draft recommendations.

### Stage 1: in‐person voting

The purpose of the voting in stage 1 was to screen out recommendations that were not viable in LMICs, and to identify recommendations that required rewording to improve their clarity and global relevance. Voting was conducted in person during a dedicated session at an international global surgery research meeting held in Kigali, Rwanda, on 1 November 2018. This meeting was attended by surgeons, anaesthetists and research methodologists. All attendees were invited to participate in voting; however, only responses from LMIC clinicians were included in the final analysis.

Each draft recommendation was voted on in turn. Rather than focusing on current availability, participants were asked to vote on whether, assuming that necessary materials were available, implementation in routine clinical care of each recommendation would be feasible in LMIC settings. Voting was conducted using a mobile platform that allowed votes to be submitted anonymously. Participants received immediate results following each vote. This feedback informed discussion of whether the recommendation required rewording before moving on to voting on the next recommendation. Discussion was chaired by a senior LMIC surgeon and a HIC surgeon with experience of producing national guidelines.

Following conclusion of the Kigali meeting, a subgroup reviewed all recommendations in light of the discussion feedback. Recommendations supported by fewer than half the LMIC clinicians were preplanned to be eliminated unless subsequent discussion identified how they might be reworded to increase their acceptability. If recommendations were supported by more than half the LMIC surgeons, but were found to be ambiguous, they were redrafted to ensure clarity and acceptability. If it was not possible to redraft recommendations to address feedback, they were eliminated by the committee. The revised list of recommendations was taken forward to the second stage.

### Stage 2: online voting

Consultant (attending) surgeons, representing LMICs, who had participated in the in‐person voting were invited to participate in the second stage and were e‐mailed a link to an online survey. To ensure balance in country representation, two senior Rwandan surgeons were invited to participate from among the Rwandan surgeons who had participated in the in‐person voting. The survey was open from 8 to 18 November 2018. Participants were asked three binary questions about each of the revised recommendations:

- Is this your current practice (yes or no)?
- Is this recommendation appropriate to your setting (yes or no)?
- Would implementing this recommendation in your setting be easy or difficult?

Respondents were invited to enter free‐text comments to justify their decisions. Recommendations identified as appropriate by fewer than 75 per cent of participants were preplanned to be eliminated, unless it was possible to reword them to address concerns raised in free‐text comments.

### Search for ongoing research

A search of the WHO International Clinical Trials Registry Platform was undertaken to identify ongoing RCTs relating to the recommendations being taken forward to the third stage. The search was completed on 5 June 2019 using the keywords ‘wound infection’ and ‘surgical site infection’. Only RCTs with sample sizes greater than 150 patients were included.

### Stage 3: in‐person meeting of guideline panel

An international guideline panel met in person on 17 June 2019 in Dubai, United Arab Emirates. The panel included senior surgeons and methodologists. The panel's deliberations were led by the same individuals who had co‐chaired discussion in stage 1. The results of the voting in stage 2 informed the guideline panel's discussions. For each draft recommendation the panel formed a consensus on which of the following decisions to take:

- To accept it with its current wording.
- To accept it after rewording aimed at either reducing its ambiguity, or maximizing its relevance. When appropriate, the panel combined separate draft statements into single recommendations.
- To change it to a research recommendation. Research recommendations were made if relevant ongoing trials were identified, and the panel agreed that the outcome of these trials should be awaited before making a recommendation. In addition, if the panel agreed that it was not possible to make a decision based on the existing evidence base, a recommendation was made regarding the need for future research.
- To eliminate it entirely.

Finalized recommendations were additionally stratified as: essential – the panel agreed that these recommendations are baseline measures required for safe surgery and should be implemented as a priority across all hospitals performing surgery; or desirable – the panel recognized that at present some hospitals lack resources to implement these recommendations, but agreed that all hospitals performing surgery should plan their future implementation.

## Results

### Delphi prioritization of guideline topics

**Figure 1:**
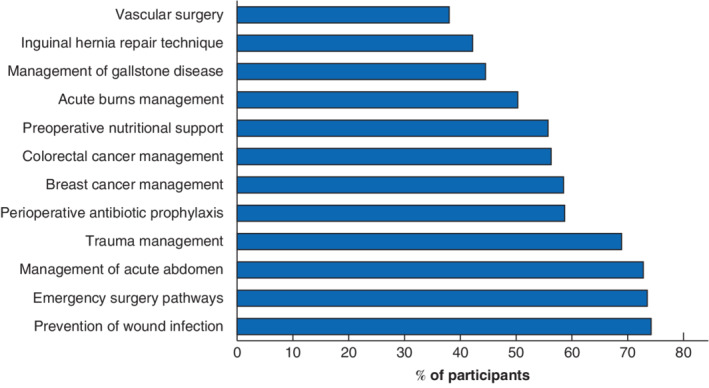
Overall ranking of top 12 shortlisted guideline topics across all low‐ and middle‐income country settings Values indicate the percentage of participants from low‐ and middle‐income countries who rated both the importance and impact of each guideline topic highly (score at least 4 of 5).

A total of 56 surgeons from across 15 LMICs proposed 34 guideline topics. The top 12 topics were shortlisted, and these were then prioritized in an online vote by 736 participants representing 82 LMICs. The overall top ranked topic (*Fig*. 1) was prevention of surgical‐site infection (SSI); this was ranked in the top two guideline topics across all country‐income strata (*Table* *S3*, supporting information). The steering committee refined the scope for this guideline to focus on preoperative and intraoperative interventions aimed at reducing SSI risk in patients undergoing abdominal surgery. Postoperative interventions and the management of SSI were not planned to be included in the guideline.

### Stage 0: preparation of initial draft recommendations

**Figure 2:**
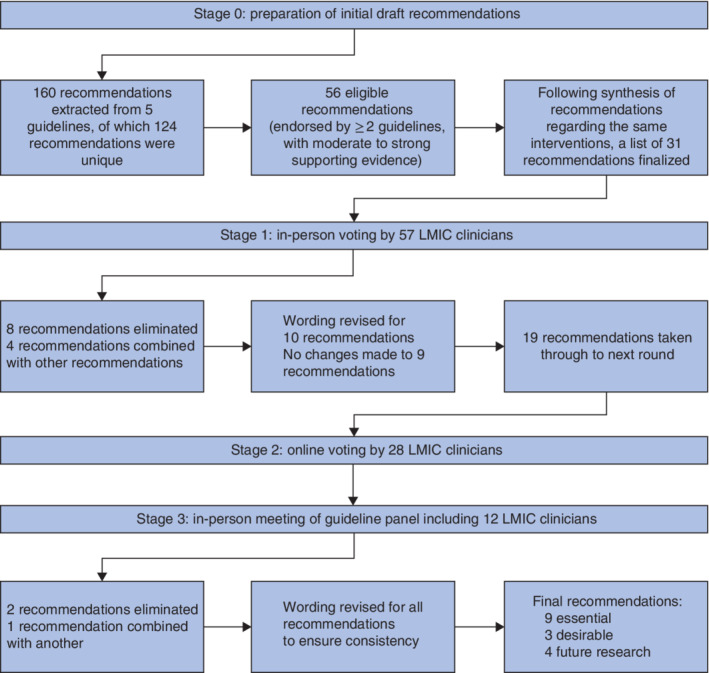
Flow of recommendations through guideline development process LMIC, low‐ and middle‐income country.

In keeping with the planned methodology, a search was completed on 12 September 2018 to identify existing guidelines relevant to the prevention of SSI. Five eligible guidelines^7^, ^8^, ^9^, ^10^, ^11^ for the prevention of SSI were identified. A total of 160 recommendations were extracted from the guidelines. Following consolidation of identical recommendations across different guidelines, 124 recommendations remained. Of these, 56 recommendations were eligible according to the criteria of being supported by at least two separate guidelines, with moderate to strong supporting evidence. Similar recommendations were synthesized and refined, producing a shortlist of 31 draft recommendations (*Table* *S2*, supporting information). The flow of recommendations through the guideline development process is summarized in *Fig*. 2.

### Stage 1: in‐person voting

**Figure 3:**
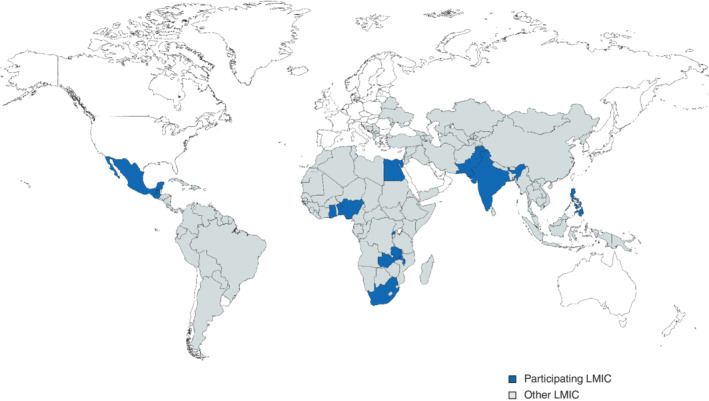
Countries represented in development of guideline for prevention of surgical‐site infection LMIC, low‐ and middle‐income country.

A total of 57 LMIC clinicians participated in the anonymous voting in Kigali. Among 45 individuals who submitted demographic data, there were 40 surgeons, three anaesthetists and two other specialists. Thirteen countries were represented (*Fig*. 3), including four low‐income countries, seven lower–middle income countries and two upper–middle‐income countries; 23 of 45 participants were Rwandan. Six of 45 of clinicians were based in rural hospitals and 38 were from public hospitals.

Of the total 31 draft recommendations, six were supported by fewer than half the participating LMIC clinicians (*Table* *S4*, supporting information). These recommendations related to methicillin‐resistant *Staphylococcus aureus* (MRSA) screening, mechanical bowel preparation for colorectal surgery, incise drapes and antibiotic‐impregnated drapes, antiseptic‐coated sutures and iodophor wound lavage. In keeping with the predefined criteria, these six recommendations were eliminated, along with another recommendation that was no longer relevant following rejection of MRSA screening. A recommendation for using either plain or antimicrobial soap was supported by 85 per cent of respondents, but subsequent discussion identified that many clinicians found this recommendation too non‐specific, so a committee decision was made to eliminate it.

Based on feedback received during discussions, the total number of recommendations was further reduced by combining four statements with other recommendations. The wording of a further ten recommendations was revised by the committee after discussion in Kigali. No changes were made to nine recommendations. The revised list of 19 recommendations proceeded to stage 2 (*Table* *S4*, supporting information).

### Stage 2: online voting

A total of 32 LMIC consultant surgeons were invited to participate in the second stage. The response rate was 88 per cent (28 of 32), with 13 countries represented. Overall, 17 of the 19 draft recommendations in stage 2 had agreement from at least 75 per cent of respondents regarding their appropriateness to LMIC settings (*Table* *S4*, supporting information). The two draft recommendations that did not reach consensus concerned omitting antibiotic prophylaxis in clean surgery, and wound edge protector devices. Several participants felt that, given high baseline SSI rates and high rates of immunosuppression (such as human immunodeficiency virus), it would be inappropriate to recommend that antibiotic prophylactic antibiotics should not be given routinely in clean surgery. Many respondents felt that the expense of wound edge protector devices in gastrointestinal surgery could not be justified, particularly given the uncertainty over their benefits.

### Stage 3: in‐person meeting of guideline panel

The guideline panel included 12 LMIC members representing ten countries, as well as five HIC members. The panel confirmed the exclusion of both draft recommendations that had been flagged in stage 2 as not being appropriate to LMIC settings. To streamline the guidelines, recommendations regarding antibiotic prophylaxis in clean‐contaminated and contaminated/dirty surgery were combined. All recommendations were reworded to ensure consistency in their format (*Table* *S5*, supporting information).

Based on a search of the WHO International Clinical Trials Registry Platform, four draft recommendations were changed to research recommendations (*Table* *S5*, supporting information). The panel agreed on a final list of nine essential and three desirable clinical recommendations (*Table* 1). It was agreed that these guidelines will be reviewed in 4 years, in 2023.

**Table 1:** Global surgery guidelines for the prevention of surgical‐site infection

Essential	Ensure patients have had a full body wash with clean water and soap before operation
	Select antibiotic prophylaxis according to published local, regional or national guidelines that takes into account expected pathogens for the operation type and local resistance patterns*
	Administer antibiotic prophylaxis to all patients undergoing clean‐contaminated, contaminated or dirty surgery*
	Administer antibiotic prophylaxis intravenously within 60 min before skin incision*
	Administer a repeat dose of antibiotic prophylaxis if the duration of operation is longer than the half‐life of the antibiotic given*
	Do not routinely continue prophylactic antibiotics beyond 24 h after operation for the purpose of reducing SSI risk*
	Ensure scrub teams decontaminate their hands before surgery using antiseptic surgical solution
	Prepare the skin at the surgical site immediately before incision, using antiseptic preparation
	During surgery under general anaesthetic, provide supplemental oxygen to maintain adequate tissue perfusion, ensuring that the patient's oxygen saturation is maintained at ≥ 95%
Desirable	If hair removal is required, do this on the operating table immediately before incision (using electric clippers if available)
	Maintain normothermia (temperature ≥ 35·5°C) during the perioperative period
	For all patients, monitor perioperative blood sugar levels and manage these according to a defined protocol
Future research	Research is required to test the effectiveness of routine antibiotic prophylaxis for reducing SSI in patients undergoing clean surgery in LMICs
	The results of ongoing research regarding the optimal solution for skin preparation should be monitored
	The results of ongoing research regarding postoperative supplemental oxygenation should be monitored
	The results of ongoing research into the effectiveness of wound lavage for prevention of SSI should be monitored

* In the context of this guideline, prophylactic antibiotics are those given specifically for the purpose of preventing the development of surgical‐site infection (SSI). This is distinct from the use of therapeutic antibiotic courses to treat established infection (such as peritonitis associated with contaminated/dirty wounds). LMIC, low‐ and middle‐income country.

## Discussion

This study has prioritized 12 topics for development as guidelines for patients needing surgical care in LMICs. The International Standards for Clinical Practice Guidelines^6^ have been used to inform the development of a novel approach to adapting existing HIC guidance collaboratively to produce a guideline that reflects the challenges of delivering surgery in LMICs, and allows health providers to prioritize implementation of key interventions that are most likely to benefit patients. This first global surgery guideline includes 12 recommendations for prevention of SSI in abdominal surgery, which have been stratified as essential or desirable to guide their implementation across all surgical settings.

All recommendations included in this document have moderate‐to‐strong supporting evidence as identified by previous guidelines. The role of the LMIC clinicians who participated in the development of this guideline was to identify likely barriers to implementation of specific recommendations, and to prioritize these based on the overall feasibility of their implementation. Surgeons representing a range of LMIC settings participated, including low‐income countries (Benin, Malawi, Rwanda, Zambia), lower–middle‐income countries (Ghana, Guatemala, Egypt, India, Nigeria, Pakistan, Philippines) and upper–middle‐income countries (Mexico, South Africa) across Africa, South Asia and Latin America. LMIC participants were regional research network leaders. Although most were not based in rural or district hospital settings, they did have substantial insights into the needs of those settings.

SSI is the commonest postoperative complication in abdominal surgery^12^, ^13^, ^14^. Initiatives to reduce it should be prioritized worldwide, but the need is greatest in LMICs, where up to one‐quarter of patients develop an SSI after gastrointestinal surgery^13^. Antimicrobial resistance is significantly more common in LMICs than HICs^13^, ^15^. The current guideline offers a potential pathway to significantly improve patient outcomes and reduce the high healthcare costs incurred by SSI^16^, in particular by providing clear guidance regarding the selection, timing and route of administration of prophylactic antibiotics. This guideline implements research findings which may reduce unnecessary antibiotic exposure at a population level, for example by reducing the duration of antibiotic prophylaxis^17^, ^18^. This will support antibiotic stewardship initiatives aimed at curbing antimicrobial resistance.

The WHO Surgical Safety Checklist has been disseminated widely, and has been demonstrated to reduce postoperative morbidity and mortality^19^, ^20^, ^21^. Some health systems have embedded an SSI bundle in local versions of the checklist to ensure that all patients receive key interventions to reduce infection risk. Surgical colleges and associations should work with healthcare providers to produce local Surgical Safety Checklists that incorporate this guideline's key recommendations. Given that the checklist is used in only half of emergency abdominal procedures in middle‐income countries and a third of procedures in low‐income countries^22^, implementation of these guidelines should be performed alongside broader initiatives to increase uptake of the checklist^23^. There remain significant evidence gaps in the prevention of SSI, highlighted by the four research recommendations made in this guideline. In order to inform policy in LMICs, it is essential that future trials incorporate health economic evaluation^24^.

## Supporting information


**Appendix S1.** Supporting Information

A PDF of the summary recommendations is available and free to download in the Supporting Information section online.
