# Supplementary material for: Delphi prioritization and development of global surgery guidelines for the prevention of surgical‐site infection
Source: Br J Surg. 2020 Mar 24;107(8):970–7. doi: 10.1002/bjs.11530 (PMC7317442; doi:10.1002/bjs.11530)
Supplement: Supplementary file 1 — Appendix S1. Supporting Information [file BJS-107-970-s001.docx]

**BJS11530**

**Delphi prioritization and development of global surgery guidelines for the prevention of surgical-site infection**

National Institute for Health Research Global Research Health Unit on Global Surgery

**Appendix 1: authorship list (PubMed citable)**

**Writing group:** Dmitri Nepogodiev (joint first author), Adewale Adisa (joint first author), Francis Atindaana Abantanga, Adesoji Ademuyiwa, Sohini Chakrabortee, Dhruva Ghosh, James Glasbey, Marie Carmela Lapitan, Ismail Lawani, Mayaba Maimbo, Rachel Moore, Dion Morton, Faustin Ntirenganya, Ahmad Uzair Qureshi, Antonio Ramos-De la Medina, Stephen Tabiri, Thomas Pinkney, Aneel Bhangu (guarantor).

**Guideline topic prioritisation (alphabetical order):** Adesoji Ademuyiwa, Anthony Adenekan, Abdus-sami Adewunmi, Adewale Adisa, Maria Lorena Aguilera, Aneel Bhangu, Bruce Biccard, Peter Brocklehurst, Sohini Chakrabortee, Ainhoa Costa, Philip Cotton, Justine Davies, Thomas M. Drake, O. James Garden, Dhruv Ghosh, James Glasbey, Parvez David Haque, Ewen M. Harrison, Jean De La Croix Allen Ingabire, Stephen R. Knight, Marie Carmela Lapitan, Ismail Lawani, Richard Lilford, Mayaba Maimbo, Janet Martin, Luis Hernandez Miguelena, Rohin Mittal, Rachel Moore, Dion Morton, Vanessa Msosa, Syed Asghar Naqi, Dmitri Nepogodiev, Faustin Ntirenganya, Jean Leon Olory-Togbe, Omar Mohamed Omar, Thomas D. Pinkney, Ahmad Uzair Qureshi, Antonio Ramos-De la Medina, Hosni Khairy Salem, Martin Smith, Sudha Sundar, Stephen Tabiri, Edwin Yenli, Raul Yepez, Eugene Zoumenou.

**Guideline development (alphabetical order):** Francis Atindaana Abantanga, Adesoji Ademuyiwa, Abdus-sami Adewunmi, Adewale Adisa, Maria Lorena Aguilera, Aneel Bhangu, Bruce Biccard, Peter Brocklehurst, Sohini Chakrabortee, Ainhoa Costa, Thomas M. Drake, Dhruva Ghosh, James Glasbey, Parvez David Haque, Ewen M. Harrison, Jean De La Croix Allen Ingabire, Conor S Jones, Chifundo Kajombo, Stephen R. Knight, Marie Carmela Lapitan, Ismail Lawani, Samuel Lawday, Mayaba Maimbo, Janet Martin, Luis Hernandez Miguelena, Rohin Mittal, Rachel Moore, Dion Morton, Vanessa Msosa, Syed Asghar Naqi, Dmitri Nepogodiev, Faustin Ntirenganya, Martin Nyundo, Jean Leon Olory-Togbe, Thomas D. Pinkney, Ahmad Uzair Qureshi, Antonio Ramos-De la Medina, Dione Parreno-Sacdalan, Hosni Khairy Salem, Martin Smith, Stephen Tabiri, Edwin Yenli, Eugene Zoumenou.

**Appendix 2: Guideline topic prioritisation**

**Methods**

A three-round Delphi exercise was conducted across the international National Institute for Health Research Global Health Research Unit on Global Surgery (NIHR-GSU) network in order to prioritise topics for essential surgical guidelines. The NIHR-GSU network at the time of this study included national research Hubs in Ghana, Mexico, Pakistan, Rwanda, and South Africa. In addition, the NIHR-GSU had a broader network of standalone research centres.

*Round 1: topic scoping*

In the first round NIHR-GSU Hub leads and other key LMIC collaborators were invited by email to submit topic proposals for global surgery guidelines. Hub leads further disseminated the topic scoping survey to anaesthetists and surgeons across their regional and national networks. Proposals were collated using an online survey open on 20 June 2018 to 10 August 2018.

*Round 2: Shortlisting*

Following closure of the electronic survey, the proposals were consolidated to remove topic duplication. As it would not be realistic to expect collaborators to score a large number of items in the subsequent voting round, a formal shortlisting process was undertaken to reduce the number of proposals. It was pre-planned for the top 12 topics to proceed to the voting round.

Shortlisting was completed independently by nine assessors, including the five Hub leads and four NIHR-GSU staff. These assessors were blinded as to the origins of proposed topics. Assessors scored each topic from 1 [lowest score] to 10 [highest score] on two criteria: (1) relevance to global surgical care and (2) potential for improving global surgical care. Total scores from the nine assessors were summed for each proposed guideline topic and the list was reviewed to ensure that a balanced set of topics proceeded to the next round.

*Round 3: Voting and ranking*

Shortlisted topics were submitted to an anonymous online vote. The voting survey was available in English, French and Spanish and was open for completion by LMIC stakeholders between 13 August 2018 and 12 September 2018. Hub leads disseminated the survey across their networks using email and WhatsApp, as well as through national surgical societies. In addition, the survey was centrally disseminated to mailing lists of active LMIC surgeons, to the WHO Global Initiative for Emergency and Essential Surgical Care list server, and also through social media.

Participants scored each topic using two criteria: (1) topic importance and (2) likely impact of guideline on this topic on patient care. Each criteria was scored from 1 [lowest score] to 5 [highest score]. Participants were recorded as supporting a multicentre research topic if they scored both importance and impact ≥4 out of 5. The proportion of participants supporting each topic was calculated.

**Results**

*Round 1: topic scoping*

A total of 56 individuals participated in topic scoping, representing 15 LMICs: Mexico (n=14), Pakistan (n=12), Ghana (n=8), Rwanda (n=4), South Africa (n=3), Benin (n=3), Nigeria (n=3), India (n=2), Egypt (n=1), Guatemala (n=1), Malawi (n=1), Peru (n=1), Philippines (n=1), Somalia (n=1), Zambia (n=1).

*Round 2: Shortlisting*

From 90 proposals, there were 34 unique topics. According to the pre-specified plan, the panel of five Hub leads and four NIHR-GSU staff shortlisted the top 12 topics. The shortlisted topics were:

- Acute burns management
- Breast cancer management
- Colorectal cancer management
- Emergency surgery pathways (service-level recommendations [e.g. emergency theatre organisation])
- Inguinal hernia repair technique
- Management of acute abdomen (patient-level recommendations [e.g. management of perforated peptic ulcer])
- Management of gallstone disease
- Perioperative antibiotic prophylaxis
- Preoperative nutritional support
- Prevention of wound infection
- Trauma management
- Vascular surgery

*Round 3: voting and ranking*

A total of 736 clinicians participated in the anonymous online voting round. Overall 82 LMICs were represented, including 31 low income countries (n=140 responses), 21 lower-middle income countries (n=357), and 30 upper-middle income countries (n=239).

The top three topics were ‘prevention of wound infection’, ‘emergency surgery patient pathways’ and ‘management of the acute abdomen’ (Figure 1). These rankings were broadly consistent across country income strata (Table S2).

**Table S1: Adherence to the components of the International Standards for Clinical Practice Guidelines**

| **Component** | **Adherence** |
| --- | --- |
| Composition of guideline development group | The guideline panel developed the final recommendations (stage 3). This panel consisted of 17 individuals with representation from a diverse range of global health settings, including 10 LMICs. The panel included a range of professionals including both clinical staff and expert methodologists. A wider pool of both HIC and LMIC collaborators contributed to guideline development (stages 1 and 2). |
| Decision-making progress | The manuscript (methods) processes for ensuring transparent decision-making. The guideline panel’s deliberations at each stage of the guideline development process are reported in detail in the supplementary materials. |
| Conflicts of interest | All individuals who contributed to the development of the guideline have declared conflicts of interest. |
| Methods | The manuscript reports all relevant methods. |
| Scope of guideline | The guideline’s scope was defined prior to commencement of its development. The guideline’s scope is reported in the manuscript (methods). |
| Evidence reviews | This guideline was not intended to undertake an exhaustive literature review. Instead it is based on five other recent guidelines which were in turn developed following thorough systematic reviews. Only recommendations supported by multiple previous guidelines with moderate or strong underpinning evidence were considered for inclusion in this guideline. A search was made for relevant ongoing studies. |
| Guideline recommendations | Twelve key recommendations have been identified with each being summarised in a single sentence. Statements were extensively discussed and revised to ensure clarity. |
| Rating of evidence and recommendations | The strength of recommendations has been carefully considered with each statement rated as being either an ‘essential’ or a ‘desirable’ recommendation. Since a new literature review was not undertaken recommendations were not rated based on the reliability of the evidence. |
| Peer review | The manuscript describing the development of the guideline has been submitted for consideration by a peer-reviewed journal. |
| Guideline expiration and updating | The manuscript include the expiration date for the guideline (2023). |
| Financial support and sponsoring organisation | The financial support for the development of this guideline has been reported in the manuscript. |

**Table S2: Initial formulation of draft recommendations based on published guidelines**

| **Topic** | **WHO** | **CDC** | **NICE** | **SHEA** | **SIGN** | **Draft recommendations*** |
| --- | --- | --- | --- | --- | --- | --- |
| Pre-operative bathing | It is good clinical practice for patients to bathe or shower prior to surgery. | Advise patients to shower or bathe (full body) with soap (antimicrobial or non-antimicrobial) or an antiseptic agent on at least the night before the operative day. | Advise patients to shower or have a bath using soap, either the day before, or on the day of, surgery. |  |  | **A1** Patients should bathe pre-operatively.  **A2** Either plain or antimicrobial soap may be used for pre-operative bathing. |
| MRSA screening and decontamination | Treat patients with known nasal carriage of Staphylococcus aureus with perioperative intranasal applications of mupirocin 2% ointment with or without a combination of CHG body wash. |  |  | Screen for Staphylococcus aureus and decolonize surgical patients with an anti-staphylococcal agent in the preoperative setting for high-risk procedures. | Intranasal mupirocin should be used prophylactically for patients undergoing high–risk surgery who are identified with Staphylococcus aureus or MRSA. | **A3** Patients should be screened pre-operatively for MRSA.  **A4** MRSA positive patients should receive MRSA decontamination with mupirocin 2% ointment. |
| Antibiotic prophylaxis in clean surgery |  |  | Do not use antibiotic prophylaxis routinely for clean non-prosthetic uncomplicated surgery. |  | Antibiotic prophylaxis is not recommended for hernia repair or splenectomy. | **A5** Antibiotic prophylaxis should not be routinely given to patients undergoing uncomplicated clean surgery (e.g. inguinal/femoral hernia repair). |
| Antibiotic prophylaxis in clean-contaminated surgery |  |  | Give antibiotic prophylaxis to patients before clean-contaminated surgery. |  | Antibiotic prophylaxis is recommended for clean-contaminated procedures. This includes hysterectomy, liver, pancreatic, open gallbladder, bile duct surgery, oesophageal, gastric, small bowel, appendicectomy, and colorectal surgery. | **A6** Antibiotic prophylaxis should be given to all patients undergoing clean-contaminated surgery, including: gastric surgery, small bowel surgery, appendicectomy, colorectal surgery, hysterectomy. |
| Antibiotic prophylaxis in contaminated and dirty surgery |  |  | Give antibiotic prophylaxis to patients before contaminated or dirty surgery. |  |  | **A7** Antibiotic prophylaxis should be given to all patients undergoing contaminated or dirty surgery. |
| Antibiotic prophylaxis in caesarean section |  | Administer appropriate parenteral prophylactic in all caesarean section procedures. |  |  | Antibiotic prophylaxis is highly recommended for caesarean section. | **A8** Antibiotic prophylaxis should be given to all patients undergoing caesarean section. |
| Antibiotic prescribing |  | Administer preoperative antimicrobial agents only when indicated based on published clinical practice guidelines. | Use the local antibiotic formulary and always take into account the potential adverse effects when choosing specific antibiotics for prophylaxis. | Administer antimicrobial prophylaxis according to evidence-based standards and guidelines. | Antibiotics selected for prophylaxis must cover the expected pathogens for that operative site. | **A9** Antibiotic prophylaxis should be selected according to published local guidelines that take in to account expected pathogens for a given operative site and local resistance patterns. |
| Route of antibiotic administration |  |  | Consider giving a single dose of antibiotic prophylaxis intravenously on starting anaesthesia. |  | Intravenous prophylactic antibiotics should be given. | **A10** Antibiotic prophylaxis should be administered intravenously. |
| Timing of antibiotic administration |  | Administer preoperative antimicrobial agents such that a bactericidal concentration of the agents is established in the serum and tissues when the incision is made. |  | Begin administration within 1 hour before incision to maximize tissue concentration. | Intravenous prophylactic antibiotics should be given within 60 minutes before the skin is incised and as close to time of incision as practically possible. | **A11** Antibiotic prophylaxis should be administered up to 60 minutes prior to surgical incision. |
| Duration of antibiotic prophylaxis | Do not prolong prophylactic antibiotic administration after completion of the operation for the purpose of preventing SSI. |  | Give a repeat dose of antibiotic prophylaxis when the operation is longer than the half-life of the antibiotic given. | Prophylactic antimicrobials should be re-dosed at intervals of 2 half-lives (measured from time the preoperative dose was administered) in cases that exceed this time. Discontinue agent within 24 hours after surgery. | The duration of prophylactic antibiotic therapy should be single dose except in special circumstances, for example, prolonged surgery, major blood loss. | **A12** Further prophylactic antibiotic doses should be administered in long cases or if there is excessive blood loss during the procedure.  **A13** Postoperative antibiotic prophylaxis should not routinely be continued beyond 24 hours. |
| Mechanical bowel preparation | Preoperative oral antibiotics combined with mechanical bowel preparation should be used to reduce the risk of SSI in adult patients undergoing elective colorectal surgery. Mechanical bowel preparation alone (without administration of oral antibiotics) should not be used. |  | Do not use mechanical bowel preparation routinely to reduce the risk of surgical site infection. | Use a combination of parenteral antimicrobial agents and oral antimicrobials to reduce the risk of SSI following colorectal procedures. Mechanical bowel preparation without oral antimicrobials does not decrease the risk of SSI. |  | **A14** When giving mechanical bowel preparation for colorectal procedures, it should only be given in combination with oral and intravenous antibiotic prophylaxis. |
| Preoperative hair removal | Hair should either not be removed or, if absolutely necessary, it should be removed only with a clipper. Shaving is strongly discouraged at all times. |  | Do not use hair removal routinely to reduce the risk of surgical site infection. If hair has to be removed, use electric clippers with a single-use head on the day of surgery. Do not use razors for hair removal. | Do not remove hair at the operative site unless its presence of hair will interfere with the operation. If hair removal is necessary, remove hair outside the operating room using clippers. Do not use razors. |  | **A15** Avoid removing body hair preoperatively unless required.  **A16** If preoperative hair removal is needed use electric clippers.  **A17** If preoperative hair removal is needed do not shave using a razor. |
| Surgical scrubbing | Surgical hand preparation should be performed by scrubbing with either a suitable antimicrobial soap and water or suitable alcohol-based hand rub before donning sterile gloves. |  | The operating team should wash their hands prior to the first operation on the list using an aqueous antiseptic surgical solution, with a single-use brush or pick for the nails, and ensure that hands and nails are visibly clean. |  |  | **A18** Surgeons should decontaminate their hands using antiseptic surgical solution. |
| Skin preparation | The panel recommends alcohol-based antiseptic solutions based on CHG for surgical site skin preparation in patients undergoing surgical procedures. | Perform intraoperative skin preparation with an alcohol-based antiseptic agent unless contraindicated. | Prepare the skin at the surgical site immediately before incision using an antiseptic preparation. Consider using an alcohol-based solution of chlorhexidine. | Use alcohol-containing preoperative skin preparatory agents if no contraindication exists. |  | **A19** Prepare the skin at the surgical site immediately prior to incision using antiseptic preparation.  **A20** Use an alcohol-based antiseptic to prepare the skin. |
| Drapes | The panel suggests not to use plastic adhesive incise drapes with or without antimicrobial properties for the purpose of preventing SSI. | The use of plastic adhesive drapes with or without antimicrobial properties is not necessary for the prevention of SSI. |  | Do not routinely use antiseptic drapes as a strategy to prevent SSIs. |  | **A21** Do not use plastic incise drapes.  **A22** Do not use antiseptic impregnated drapes. |
| Normothermia | Use warming devices in the OR and during the surgical procedure for patient body warming. | Maintain perioperative normothermia |  | Maintain normothermia (temperature of 35.5°C or more) during the perioperative period |  | **A23** Maintain normothermia (temperature ≥35.5 degrees) during the perioperative period |
| Glycaemic control | Use of protocols for intensive perioperative blood glucose control for both diabetic and non-diabetic adult patients. | Implement perioperative glycaemic control and use blood glucose target levels less than 200 mg/dL in patients with and without diabetes. |  |  |  | **A24** Monitor blood sugar levels peri-operatively and manage these according to a defined protocol. |
| Peri-operative oxygenation | Adult patients undergoing general anaesthesia with endotracheal intubation should receive an 80% fraction of inspired oxygen intraoperatively and, if feasible, in the immediate postoperative period for 2-6 hours. | For patients with normal pulmonary function undergoing general anaesthesia with endotracheal intubation, administer increased FIO_2_ during surgery and after extubation in the immediate postoperative period. | Maintain optimal oxygenation during surgery. In particular, give patients sufficient oxygen during major surgery and in the recovery period to ensure that a haemoglobin saturation of more than 95% is maintained. | Optimize tissue oxygenation by administering supplemental oxygen during and immediately following surgical procedures involving mechanical ventilation. |  | **A25** Provide supplemental oxygen during surgery to maintain adequate perfusion, ensuring haemoglobin saturation ≥95% is maintained.  **A26** Provide supplemental oxygen immediately following surgery. |
| Fluid management | Use goal-directed fluid therapy intraoperatively. | To optimize tissue oxygen delivery, maintain adequate volume replacement. |  | Supplemental oxygen is most effective when combined with appropriate volume replacement. |  | **A27** Maintain adequate volume replacement |
| Wound edge protectors | Consider the use of wound protector devices in clean-contaminated, contaminated and dirty abdominal surgical procedures. |  |  | Use impervious plastic wound protectors for gastrointestinal and biliary tract surgery. |  | **A28** Use a wound edge protector device in gastrointestinal and biliary surgery. |
| Wound lavage | Consider the use of irrigation of the incisional wound with an aqueous PVP-I solution before closure, particularly in clean and clean-contaminated wounds | Consider intraoperative irrigation of deep or subcutaneous tissues with aqueous iodophor solution. | Do not use wound irrigation to reduce the risk of surgical site infection. | Perform antiseptic wound lavage. |  | **A29** Perform wound lavage.  **A30** Use idophor (e.g. betadine) solution for wound lavage. |
| Triclosan sutures | Use triclosan-coated sutures, independent of the type of surgery | Consider the use of triclosan-coated sutures. | Consider using triclosan-coated sutures. | Do not routinely use antiseptic-impregnated sutures as a strategy to prevent SSIs. |  | **A31** Use antiseptic-impregnated (e.g. triclosan) sutures. |

CDC: Centers for Disease Control and Prevention, NICE: National Institute for Health and Care Excellence, SHEA: Society for Healthcare Epidemiology of America, SIGN: Scottish Intercollegiate Guidelines Network, WHO: World Health Organization

*Recommendations were longlisted if they were supported by two or more guidelines, with one or more guidelines having reported moderate or strong evidence to support the recommendation. For example, recommendations regarding timing of antibiotic administration were made by CDC (category 1B: strong recommendation based on accepted practice), SHEA (quality of evidence grade I – high quality evidence based on a wide range of studies), and SIGN (recommendation grade B – based on a body of high quality case control or cohort studies or systematic reviews) guidelines. Therefore, as three guidelines made a recommendation on timing of antibiotic administration, with two guidelines reporting moderate to strong supporting evidence, this was longlisted. Since different guidelines can make slightly different recommendations regarding the same interventions (as in the timing of antibiotic prophylaxis example), similar recommendations were combined to produce a streamlined draft recommendations.

**Table S3: Ranking of top 12 shortlisted guideline topics, stratified by country income**

| **Low income country respondents** | | |  | **Lower-middle income country respondents** | | |  | **Upper-middle income country respondents** | | |
| --- | --- | --- | --- | --- | --- | --- | --- | --- | --- | --- |
| Rank | Short topic title | Score |  | Rank | Short topic title | Score |  | Rank | Short topic title | Score |
| 1 | Emergency surgery pathways* | 77.7% |  | 1 | Prevention of wound infection | 75.3% |  | 1 | Emergency surgery pathways* | 73.0% |
| 2 | Prevention of wound infection | 74.8% |  | 2 | Management of acute abdomen† | 73.9% |  | 2 | Prevention of wound infection | 72.3% |
| 3 | Management of acute abdomen† | 71.9% |  | 3 | Emergency surgery pathways* | 72.3% |  | 3 | Management of acute abdomen† | 71.6% |
| 4 | Trauma management | 70.7% |  | 4 | Trauma management | 71.4% |  | 4 | Colorectal cancer management | 64.6% |
| 5 | Acute burns management | 57.9% |  | 5 | Breast cancer management | 66.4% |  | 5 | Trauma management | 64.0% |
| =5 | Peri-operative antibiotic prophylaxis | 57.9% |  | 6 | Peri-operative antibiotic prophylaxis | 58.0% |  | 6 | Peir-operative antibiotic prophylaxis | 60.1% |
| 7 | Preoperative nutritional support | 54.7% |  | 7 | Colorectal cancer management | 55.5% |  | 7 | Preoperative nutritional support | 57.6% |
| 8 | Breast cancer management | 50.4% |  | 8 | Preoperative nutritional support | 54.9% |  | 8 | Management of gallstone disease | 52.9% |
| 9 | Colorectal cancer management | 44.2% |  | 9 | Acute burns management | 50.3% |  | 9 | Breast cancer management | 51.5% |
| 10 | Management of gallstone disease | 39.3% |  | 10 | Inguinal hernia repair technique | 41.6% |  | =10 | Acute burns management | 45.8% |
| 11 | Inguinal hernia repair technique | 37.4% |  | 11 | Management of gallstone disease | 40.9% |  | =10 | Inguinal hernia repair technique | 45.8% |
| 12 | Vascular surgery | 35.3% |  | 12 | Vascular surgery | 36.1% |  | 12 | Vascular surgery | 42.3% |

*Service-level recommendations (e.g. emergency theatre organisation)

†Patient-level recommendations (e.g. management of perforated peptic ulcer)

**Table S4: Results of stage 1 voting and discussion on original recommendations, and results of stage 2 voting on revised recommendations**

| **Draft recommendation (considered in stage 1)** | **Stage 1**  **Voting (score)** | **Discussion at Stage 1** | **Revised recommendation (considered in stage 2)** | **Stage 2 voting** | | |
| --- | --- | --- | --- | --- | --- | --- |
|  |  |  |  | **CP** | **AS** | **EI** |
| **A1** Patients should bathe pre-operatively. | 90% | The uncertainty over the optimal timing of bathing (day before surgery or on the morning of surgery) was discussed and it was noted that guidelines make mixed recommendations regarding this. | **B1** Patients should bathe pre-operatively | 60% | 95% | 75% |
| **A2** Either plain or antimicrobial soap may be used for pre-operative bathing. | 85% | Although there was high initial agreement, in the discussion it was highlighted that as no specific soap was recommended it was unclear that this recommendation would be helpful in clinical practice. | Recommendation eliminated (committee decision) |  |  |  |
| **A3** Patients should be screened pre-operatively for MRSA. | 27% | Routine screening was felt to be unnecessary due to perceived low prevalence of MRSA in elective patients. Routine screening felt to be impractical and uneconomical. | Recommendation eliminated (Round 1 agreement <40%) |  |  |  |
| **A4** MRSA positive patients should receive MRSA decontamination with mupirocin 2% ointment. | - | This recommendation was not discussed due to elimination of recommendation A3. | Recommendation eliminated |  |  |  |
| **A5** Antibiotic prophylaxis should not be routinely given to patients undergoing uncomplicated clean surgery (e.g. inguinal/femoral hernia repair). | 71% | Concerns were noted regarding the high baseline SSI rates in clean surgery in LMICs. Several participants felt antibiotic prophylaxis to be frequently warranted. Further examples of clean surgery were requested. | **B2** Antibiotic prophylaxis should NOT be routinely given to patients undergoing clean surgery, including: uncomplicated inguinal hernia repair with mesh, adhesiolysis | 30% | 70% | 65% |
| **A6** Antibiotic prophylaxis should be given to all patients undergoing clean-contaminated surgery, including: gastric surgery, small bowel surgery, appendicectomy, colorectal surgery, hysterectomy. | 90% | It was noted that some surgical teams may need to be trained to ensure they appreciate the full scope of clean-contaminated surgery- e.g. that this includes bile duct surgery and hysterectomy. | **B3** Antibiotic prophylaxis should be given to patients undergoing clean-contaminated surgery, including: gastric surgery, small bowel surgery, appendicectomy, colorectal surgery, biliary surgery, hysterectomy, and caesarean section | 100% | 95% | 90% |
| **A7** Antibiotic prophylaxis should be given to all patients undergoing contaminated or dirty surgery. | 93% | There were no specific objections to this recommendation. | **B4** Antibiotic prophylaxis should be given to patients undergoing contaminated or dirty surgery | 100% | 100% | 95% |
| **A8** Antibiotic prophylaxis should be given to all patients undergoing caesarean section. | 85% | In light of the clarification in recommendation B3 that clean-contaminated surgery includes caesarean section, these recommendations were combined. | Combined with recommendation B3 |  |  |  |
| **A9** Antibiotic prophylaxis should be selected according to published local guidelines that take in to account expected pathogens for a given operative site and local resistance patterns. | 91% | There were no specific objections to this recommendation. | **B5** Antibiotic prophylaxis should be selected according to published local guidelines that take in to account expected pathogens for that operation type and local resistance patterns | 70% | 100% | 70% |
| **A10** Antibiotic prophylaxis should be administered intravenously. | 96% | Reservations were noted regarding the cost-effectiveness of intravenous antibiotics in LMIC settings. | **B6** Antibiotic prophylaxis should be administered intravenously within 60 minutes before the skin is incised | 95% | 100% | 90% |
| **A11** Antibiotic prophylaxis should be administered up to 60 minutes prior to surgical incision. | 90% | It was identified that as this recommendation assumed an intravenous route for antibiotic administration, so it was combined with the recommendation for intravenous antibiotic use. The timing (pre- or post-incision) was discussed. | Combined with recommendation B6 |  |  |  |
| **A12** Further prophylactic antibiotic doses should be administered in long cases or if there is excessive blood loss during the procedure. | 76% | The combination of two distinct clinical scenarios in one recommendation was felt to be confusing. There was uncertainty as to what would qualify as a “long case”. | **B7** A repeat dose of antibiotic prophylaxis should be administered when the operation is longer than the half-life of the antibiotic given | 75% | 100% | 90% |
| **A13** Postoperative antibiotic prophylaxis should not routinely be continued beyond 24 hours. | 90% | The word “prophylaxis” was felt by many participants to imply a single dose, making the recommendation’s wording confusing. | **B8** Antibiotics should not routinely be continued beyond 24 hours post-operatively for the purpose of reducing SSI risk | 70% | 95% | 90% |
| **A14** When giving mechanical bowel preparation for colorectal procedures, it should only be given in combination with oral and intravenous antibiotic prophylaxis. | 36% | It was felt that this recommendation was targeted at a very specific patient group and the full detail of bowel preparation was therefore outside of the scope of this guideline. | Recommendation eliminated (Round 1 agreement <40%) |  |  |  |
| **A15** Avoid removing body hair preoperatively unless required. | 94% | There was uncertainty as to what “unless required” meant. The timing of hair removal (preoperative versus on-table) was discussed. | **B9** If hair removal is required, this should be done on the operating table immediately prior to incision, using electric clippers (if available) | 65% | 80% | 70% |
| **A16** If preoperative hair removal is needed use electric clippers. | 71% | A lack of availability of electric clippers in many settings was highlighted. | Combined with recommendation B9 |  |  |  |
| **A17** If preoperative hair removal is needed do not shave using a razor. | 70% | A preference for electric clippers was felt to imply that shaving with a razor should be avoided, making a specific recommendation on shaving unnecessary. | Combined with recommendation B9 |  |  |  |
| **A18** Surgeons should decontaminate their hands using antiseptic surgical solution. | 78% | A lack of availability of antiseptic surgical solution in some settings was highlighted. | **B10** Surgeons should decontaminate their hands prior to surgery using antiseptic surgical solution | 90% | 100% | 95% |
| **A19** Prepare the skin at the surgical site immediately prior to incision using antiseptic preparation. | 98% | There were no specific objections to this recommendation. | **B11** Prepare the skin at the surgical site immediately prior to incision, using antiseptic preparation | 95% | 100% | 100% |
| **A20** Use an alcohol-based antiseptic to prepare the skin. | 63% | Many participants expressed uncertainty over the superiority of alcohol-based preparations over chlorhexidine-based preparations in their settings. | **B12** Use an alcohol-based antiseptic to prepare the skin | 80% | 95% | 95% |
| **A21** Do not use plastic incise drapes. | 26% | The evidence base for incise drapes was felt to be weak in LMIC settings. | Recommendation eliminated (Round 1 agreement <40%) |  |  |  |
| **A22** Do not use antiseptic impregnated drapes. | 38% | The evidence base for antiseptic impregnated drapes was felt to be weak in LMIC settings. | Recommendation eliminated (Round 1 agreement <40%) |  |  |  |
| **A23** Maintain normothermia (temperature ≥35.5 degrees) during the perioperative period | 98% | There were no specific objections to this recommendation. | **B13** Maintain normothermia (temperature ≥35.5 degrees) during the perioperative period | 75% | 100% | 90% |
| **A24** Monitor blood sugar levels peri-operatively and manage these according to a defined protocol. | 90% | There were no specific objections to this recommendation. | **B14** Monitor blood sugar levels perioperatively and manage these according to a defined protocol | 65% | 95% | 80% |
| **A25** Provide supplemental oxygen during surgery to maintain adequate perfusion, ensuring haemoglobin saturation ≥95% is maintained. | 98% | It was questioned whether this recommendation should apply to all surgical procedures or only those performed under general anaesthetic. | **B15** During surgery with general anaesthetic, provide supplemental oxygen to maintain adequate perfusion, ensuring oxygen saturation ≥95% is maintained | 85% | 100% | 100% |
| **A26** Provide supplemental oxygen immediately following surgery. | 63% | The duration of postoperative supplemental oxygenation was discussed. | **B16** To reduce risk of SSI, immediately following surgery with general anaesthetic, provide supplemental oxygen for 2-6 hours | 35% | 90% | 70% |
| **A27** Maintain adequate volume replacement | 100% | It was questioned whether this recommendation should apply to all surgical procedures or only those performed under general anaesthetic. | **B17** During surgery with general anaesthesia maintain adequate circulating volume, to reduce risk of SSI | 95% | 100% | 95% |
| **A28** Use a wound edge protector device in gastrointestinal and biliary surgery. | 64% | There was a concern that for this relatively expensive device there is a lack of cost-effectiveness data from LMIC settings. | **B18** Use a wound edge protector device in gastrointestinal and biliary surgery | 10% | 50% | 25% |
| **A29** Perform wound lavage. | 75% | There were no specific objections to this recommendation. | **B19** Perform wound lavage at the time of wound closure | 80% | 100% | 100% |
| **A30** Use idophor (e.g. betadine) solution for wound lavage. | 22% | The evidence base for used of specific solutions was felt to be weak. | Recommendation eliminated (Round 1 agreement <40%) |  |  |  |
| **A31** Use antiseptic-impregnated (e.g. triclosan) sutures. | 31% | There was a concern that for this relatively expensive device there is a lack of cost-effectiveness data from LMIC settings. | Recommendation eliminated (Round 1 agreement <40%) |  |  |  |

Stage 2 voting results present the proportion of participants reporting each recommendation to be current practice in their setting [CP], appropriate to their setting [AS], to be easy to implement in their setting [EI].

**Table S5: Stage 3 guideline panel discussion and final recommendations**

| **Draft recommendation considered in stage 3** | **Guideline panel discussion** | **Decision** | **Final recommendation** |
| --- | --- | --- | --- |
| **B1** Patients should bathe pre-operatively. | - The recommendation to “bathe” was noted as potentially suggesting that patients should have a bath. Many patients would not have access to a bath. Therefore re-worded to use term “wash” which could include bucket bath. - Challenges with patients not having access to clean water for washing discussed. Agreed that removal of gross contamination would be helped by using soap. - Concerns regarding lack of warm water in winter and affordability of soap were noted bu felt to be major considerations. | Recommendation re-worded; classified as essential. | **E1** Ensure patients have had a full body wash pre-operatively with clean water and soap. |
| **B2** Antibiotic prophylaxis should NOT be routinely given to patients undergoing clean surgery, including: uncomplicated inguinal hernia repair with mesh, adhesiolysis. | - The need to avoid unnecessary use of antibiotics to reduce spread of antimicrobial resistance was noted. - Rationales for using prophylaxis routinely in clean surgery included high baseline infection rates in clean surgery and high rates of immunosuppression (e.g. HIV, diabetes) amongst surgical patients. This was a particular concern in clean procedures where a prosthesis (e.g. hernia mesh) is implanted. - Current evidence base was thought to be primarily from high income countries so given differences in baseline patient characteristic between HICs/LMICs It was felt that specific studies are needed to explore this in LMICs. - It was considered unrealistic to propose this recommendation given that it is unlikely to be accepted by many surgeons who would be concerned that SSI rates would increase if antibiotic prophylaxis is not given. | Changed to research recommendation. | **R1** Research is required to test the effectiveness of routine antibiotic prophylaxis for reducing surgical site infection in patients undergoing clean surgery in LMICs. |
| **B3** Antibiotic prophylaxis should be given to patients undergoing clean-contaminated surgery, including: gastric surgery, small bowel surgery, appendicectomy, colorectal surgery, biliary surgery, hysterectomy, and caesarean section |  | Recommendation merged with B4; classified as essential. |  |
| **B4** Antibiotic prophylaxis should be given to patients undergoing contaminated or dirty surgery | - To simplify guidance it was agreed to combine recommendations B3 and B4. - It was agreed that all patients having clean-contaminated, contaminated, or dirty surgery should have a prophylactic antibiotic dose, but it was recognised that clarity is required in the final document that some patients require longer treatment courses, for example, if they have established peritonitis. | Recommendation re-worded; classified as essential. | **E3** Administer antibiotic prophylaxis to all patients undergoing clean-contaminated, contaminated, or dirty surgery. |
| **B5** Antibiotic prophylaxis should be selected according to published local guidelines that take in to account expected pathogens for that operation type and local resistance patterns | - It was noted that some centres lack microbiology expertise and laboratory facilities to culture organisms and track resistance patterns. In these circumstances, although local guidelines are preferable when available, it was felt appropriate to use regional or national guidelines. | Recommendation re-worded; classified as essential. | **E2** Select antibiotic prophylaxis according to published local, regional, or national guidelines that take into account expected pathogens for the operation type and local resistance patterns. |
| **B6** Antibiotic prophylaxis should be administered intravenously within 60 minutes before the skin is incised |  | Recommendation accepted; classified as essential. | **E4** Administer antibiotic prophylaxis intravenously within 60 minutes before skin incision. |
| **B7** A repeat dose of antibiotic prophylaxis should be administered when the operation is longer than the half-life of the antibiotic given | - Alternative wording that would stipulate a specific time point (e.g. 90 minutes) beyond which a repeat antibiotic dose should be given. | Recommendation accepted; classified as essential. | **E5** Administer a repeat dose of antibiotic prophylaxis if the duration of operation is longer than the half-life of the antibiotic given. |
| **B8** Antibiotics should not routinely be continued beyond 24 hours post-operatively for the purpose of reducing SSI risk | - Noted that it is important to ensure that there is clarity that this recommendation relates to antibiotics administered for prophylactic purposes only. The final document should be clear that some patients require longer treatment courses for established infections. - Use of prolonged antibiotic courses was felt to be embedded in many clinicians’ practices. There were concerns regarding training that junior doctors receive in antibiotic prescribing, but may be addressed by publication of guidelines. | Recommendation re-worded; classified as essential. | **E6** Do not routinely continue prophylactic antibiotics beyond 24 hours post-operatively for the purpose of reducing SSI risk. |
| **B9** If hair removal is required, this should be done on the operating table immediately prior to incision, using electric clippers if available | - Greatest concern regarding this recommendation is the limited availability of high quality electric clippers in LMIC settings. Agreement that the main emphasis of the recommendation should be on timing of hair removal rather than method. - Cultural barriers to implementation were discussed; for example, some patients may shave with a blade prior to coming to hospital. However, this could be addressed by counselling patients in the pre-operative setting. | Recommendation re-worded; classified as desirable. | **D1** If hair removal is required, do this on the operating table immediately prior to incision (using electric clippers if available). |
| **B10** Surgeons should decontaminate their hands prior to surgery using antiseptic surgical solution | - It was recognised that this recommendation is relevant to all members of the scrub team (surgeon, assistant, anaesthetist, scrub nurse). - It was noted that in some settings there may be limited availability of antiseptic surgical solution, but it was agreed that decontamination of hands is essential so resources should be prioritised for this. | Recommendation re-worded; classified as essential. | **E7** Ensure scrub teams decontaminate their hands prior to surgery using antiseptic surgical solution. |
| **B11** Prepare the skin at the surgical site immediately prior to incision, using antiseptic preparation |  | Recommendation accepted; classified as essential. | **E8** Prepare the skin at the surgical site immediately prior to incision, using antiseptic preparation. |
| **B12** Use an alcohol-based antiseptic to prepare the skin | - The additional cost and/or reduced availability of alcohol-based solutions was noted. - It was felt that there is a lack of evidence to support this recommendation in LMIC settings. - Given that there are multiple ongoing trials in both HIC and LMIC settings recruiting almost 20,000 patients, it was agreed that a research recommendation was appropriate. | Changed to research recommendation. | **R2** The results of ongoing research regarding optimal solution for skin preparation should be monitored and skin preparation recommendations reviewed in light of this. |
| **B13** Maintain normothermia (temperature ≥35.5 degrees) during the perioperative period | - It was agreed that this recommendation would be challenging to implement in some settings where warmers are not available. | Recommendation accepted; classified as desirable. | **D2** Maintain normothermia (temperature ≥35.5 degrees) during the perioperative period. |
| **B14** Monitor blood sugar levels perio-peratively and manage these according to a defined protocol | - The recommendation was re-worded to clarify that blood sugar levels should be monitored for all patients, not just diabetic patients. - Management of high blood sugar levels was felt to be beyond the scope of this guideline, though all hospitals should have a protocol in place for this. - This recommendation may be challenging to implement in some settings where glucose monitors are not available. | Recommendation re-worded; classified as desirable. | **D3** For all patients, monitor blood sugar levels peri-operatively and manage these according to a defined protocol. |
| **B15** During surgery with general anaesthetic, provide supplemental oxygen to maintain adequate perfusion, ensuring oxygen saturation ≥95% is maintained | - The recommendation was re-worded to clarify that the oxygen saturation refers to (patient) SpO_2_ rather than the concentration of oxygen being delivered. - It was noted that some theatres have limited availability of pulse oximeters, but it was agreed that monitoring of oxygen saturations is essential, so resources should be prioritised for this. | Recommendation accepted; classified as essential. | **E9** During surgery with general anaesthetic, provide supplemental oxygen to maintain adequate tissue perfusion, ensuring patients’ oxygen saturation is maintained ≥95%. |
| **B16** To reduce risk of SSI, immediately following surgery with general anaesthetic, provide supplemental oxygen for 2-6 hours | - There are multiple challenges in implementing this recommendation given that in many recovery rooms there is insufficient monitoring equipment and insufficient oxygen supplies/ concentrators on the ward. - It was noted that a major international randomised trial is planned in LMICs that will include evaluation of postoperative supplemental oxygenation. | Changed to research recommendation. | **R3** The results of ongoing research regarding postoperative supplemental oxygenation should be monitored and the recommendation reviewed light of this. |
| **B17** During surgery with general anaesthesia maintain adequate circulating volume, to reduce risk of SSI | - The recommendation was felt to be too non-specific regarding what constitutes ‘adequate circulating volume’ and how this should be maintained. - It was felt that considerations regarding peri-operative fluid management are multifactorial with prevention of SSI only one factor, so a more holistic approach for fluid management is required that is beyond the scope of this guideline. - Overall, it was felt the recommendation was impractical. | Recommendation eliminated |  |
| **B18** Use a wound edge protector device in gastrointestinal and biliary surgery | - It was noted that there is limited access to wound edge protector devices, particularly in view of the high cost. It was felt hospitals are unlikely to invest in a device that is thought to be not cost-effective. - It was noted that there is no strong evidence to support use of wound edge protector devices. | Recommendation eliminated |  |
| **B19** Perform wound lavage at the time of wound closure | - The recommendation was felt to be too broad since lavage may not be appropriate to all patients. - It was unclear how the recommendation should be implemented since the solution type for lavage was not specified. - It was noted that there are two ongoing randomised trials evaluation wound lavage. | Changed to research recommendation. | **R4** The results of ongoing research in to the effectiveness of wound lavage for prevention of surgical site infection should be monitored. |
