# Supplementary material for: Delphi prioritization and development of global surgery guidelines for the prevention of surgical‐site infection
Source: Br J Surg. 2020 Mar 24;107(8):970–7. doi: 10.1002/bjs.11530 (PMC7317442; doi:10.1002/bjs.11530)
Supplement: Supplementary file 2 — A PDF of the summary recommendations is available and free to download in the Supporting Information section online. [file BJS-107-970-s002.pdf]

Preventing Surgical-Site Infections

BJS

DOI: 10.1002/bjs.11530

Preoperative

- Full body wash
- Prepare surgical site immediately before incision
- Decontaminate hands
- *Remove hair on table**
  (with electric clippers if available)

Perioperative

- Maintain saturation ≥ 95%
- *Monitor & correct blood glucose**
- *Maintain normothermia**

Antibiotics

- Use prophylactically for clean-contaminated, contaminated or dirty surgery
- Select according to guidelines
  based on operation and local resistance patterns
- Administer i.v. within 60 minutes before incision
- Repeat dose if operation longer than half-life of antibiotic
- Do not routinely continue beyond 24 hours

☐ Essential
⊠ Desirable

https://NIHRGlobalSurgery.org/guidelines

NIHR Global Health Research Unit on
Global Surgery
